# Formation of complex hydrocarbon systems from methane at the upper mantle thermobaric conditions

**DOI:** 10.1038/s41598-020-61644-5

**Published:** 2020-03-12

**Authors:** Aleksandr Serovaiskii, Vladimir Kutcherov

**Affiliations:** Gubkin Russian State University of Oil and Gas (National Research University), Department of Physics, Leninsky avenue 65/1, Moscow, 119991 Russia

**Keywords:** Biogeochemistry, Carbon cycle, Chemistry, Organic chemistry

## Abstract

The existence of methane in the Earth’s mantle does not cause any doubt, however, its possible chemical transformation under the mantle thermobaric conditions is not enough known. Investigation of methane at the upper mantle thermobaric conditions, using diamond anvil cells, demonstrated the possible formation of ethane, propane and n-butane from methane, however, theoretical calculations of methane behaviour at extreme temperature and pressure predicted also heavier hydrocarbons. We experimentally investigated the chemical transformations of methane at the upper mantle thermobaric conditions, corresponding to the depth of 70–80 km (850–1000 K, 2.5 GPa), using “Toroid”-type Large reactive volume device and gas chromatography. The experimental results demonstrated the formation of the complex hydrocarbon mixture up to C_7_ with linear, branched and cycled structures and benzene. Unsaturated hydrocarbons were detected on the trace level in the products mixture. The increasing of exposure time leaded to growth of heavier components in the product systems. The data obtained suggest possible existence of complex hydrocarbon mixtures at the upper mantle thermobaric conditions and provide a new insight on the possible pathways of the hydrocarbons synthesis from methane in the upper mantle.

## Introduction

As the simplest saturated hydrocarbon, methane plays a significant role in global life. Methane is the most abundant organic molecule in the Universe. A vast amount of methane in connection with icy water and ammonia appears to occur in the interiors of Uranus and Neptune^1,2^. Methane infrared signals were detected in tails of comets^3,4^ and in the atmosphere of Mars^5^. Methane is an important component of the Earth’s atmosphere, being one of the greenhouse gases^6^. Methane in the Earth’s crust mostly occurs in petroleum, coal and pyroshale accumulations.

While the origin of methane in the Earth’s mantle is still debatable, its existence in mantle does not cause any doubt. Methane is seemed to be the major carbon component in the C-O-H fluid as evidenced by the composition of fumaroles^7^ and volcano gas^8,9^, and by the composition of the gaseous inclusions in diamonds^10^. Its possible formation from inorganic carbon and hydrogen components of the mantle at extreme thermobaric conditions has been experimentally demonstrated^11,12^.

The thermobaric stability of methane and its chemical transformations at extreme thermobaric conditions have always received great interest^13^. The investigation of methane chemical transformations demonstrated its decomposition into molecular hydrogen and pure carbon (in the form of soot, graphite and diamond) at severe thermobaric conditions – 10–50 GPa and 2000–3000 K^14,15^. However, the formation of heavier hydrocarbons, caused by methane polymerization, was detected at similar pressures but more moderate temperatures (above 1100 K)^16^. Kolesnikov, *et al*.^17^ detected the formation of ethane, propane and n-butane from methane at 900–1500 K and 2–5 GPa, using diamond anvil cells and Raman spectroscopy. At higher temperatures, molecular hydrogen and graphite were predominantly formed. Meanwhile, at significantly higher pressures (48 GPa), ethane and higher aliphatic hydrocarbons were detected at >1500 K^18^.

Summarizing the abovementioned information, the formation of heavier hydrocarbons from methane seems clear at the specified thermobaric conditions, and this is also confirmed by theoretical calculations^19–22^. However, while only ethane, propane and n-butane were experimentally identified in the products mixture, according to the models, heavier hydrocarbons may also be formed at extreme thermobaric conditions. The absence of more complicated hydrocarbons in the methane transformation products at extreme pressure and temperature can be possibly explained by the small amount of the sample in DAC (the most commonly used method for such experiments) and, as a result, trace amounts of heavier hydrocarbons, which are not indicated due to the limitation of the detector sensitivity. Motivated by this inference, we experimentally investigated the transformation of methane at the moderate thermobaric conditions, corresponding to the depth of 70–80 km (850–1000 K, 2.5 GPa), using a “Toroid”-type large reactive volume (LRV) device with analysis by gas chromatography.

## Experimental results

It was demonstrated that the oxidation conditions in the cavity did not influence the chemistry of the methane transformation principally^17^. Thus, in the present paper the main attention was focused on the experimental data providing the methane transformation under extreme thermobaric conditions.

**Figure 1:**
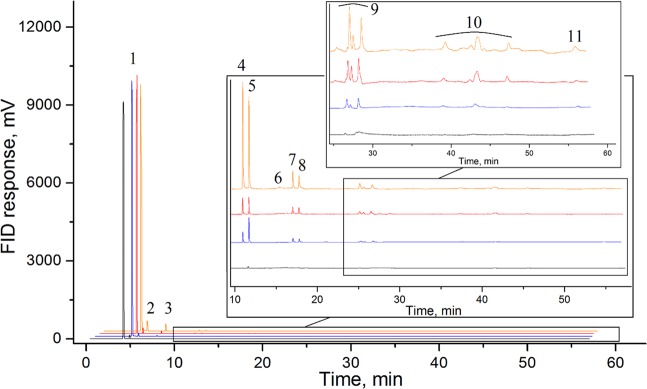
Chromatograms of the hydrocarbon products formed at 850(±25) K and 2.5(±0.2) GPa during 0.5 hours of heating (black curve), 2 hours of heating (blue curve), 4 hours of heating (red curve), and 10 hours of heating (orange curve). 1 – methane, 2 – ethane, 3 – propane, 4 – i-butane, 5 – n-butane, 6 – neo-pentane, 7 – i-pentane, 8 – n-pentane, 9 – hexanes and cyclohexane, 10 – heptanes and methyl cyclohexane, 11 – benzene.

**Figure 2:**
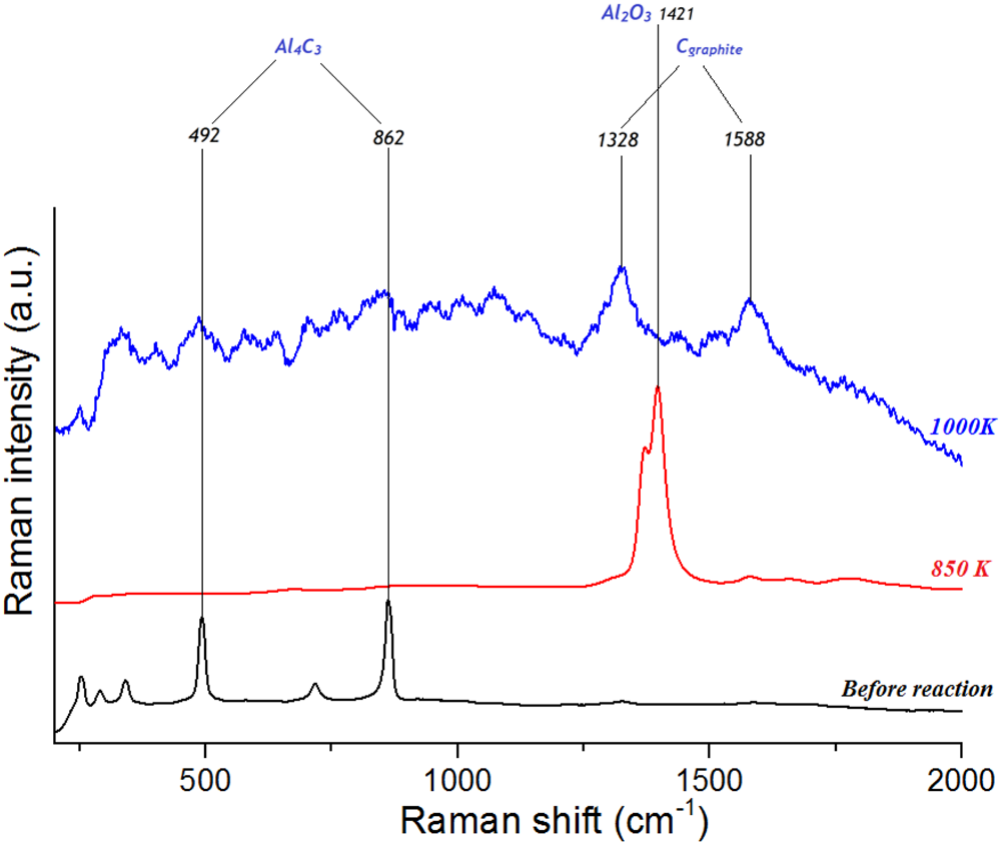
Raman spectra of the sample at ambient conditions: pure Al_4_C_3_ (black curve), the solid products formed at 850(±25) K and 2.5(±0.2) GPa during 4 hours heating (red curve), the solid products formed at 1000(±25) K and 2.5(±0.2) GPa during 4 hours heating (blue curve).

The first series of experiments were carried out at 850(±25) K and 2.5(±0.2) GPa. Methane synthesized inside the experimental cell (see Methods for more details), was heated for 0.5, 2, 4, and 10 hours at constant pressure. The gas chromatograms of the hydrocarbon products are presented in Fig. 1 (see also Supplementary Figs. 3 and 4 for more information). Alkanes from methane to heptane, both of the linear and branched structures were presented in the products mixture. Additionally, cycloalkanes (cyclohexane and methyl-cyclohexane) and benzene were detected. The fraction composition of the gaseous product mixture is presented in Table 1. Trace amounts of some light unsaturated hydrocarbons (ethylene, acetylene, propylene) were also indicated by gas chromatography (See Supplementary Fig. 5a for more details). Carbon dioxide was not generated from methane during the heating (see Supplementary Fig. 6 for more details). Raman spectra of the solid products demonstrated the presence of Al_2_O_3_ only in the mixture (Fig. 2, red curve).

**Table 1:** Composition of the products mixture after the heating (850 K, 2.5 GPa).

Component/fraction, %	*Exposure time, hours*			
	*0.5*	2	4	10
*Methane*	96.466	95.081	95.652	87.338
*Ethane*	3.125	3.935	2.401	2.457
*Propane*	0.132	0.579	1.431	2.629
*i-Butane*	0.009	0.042	0.166	0.728
*n-Butane*	0.028	0.078	0.128	0.580
*C*_5_ *fraction*	0.047	0.054	0.086	2.242
*C*_6_ *fraction*	0.063	0.091	0.058	1.406
*C*_7_ *fraction*	0.065	0.141	0.063	2.022
*Benzene*	0.007	0.041	0.014	0.597

**Figure 3:**
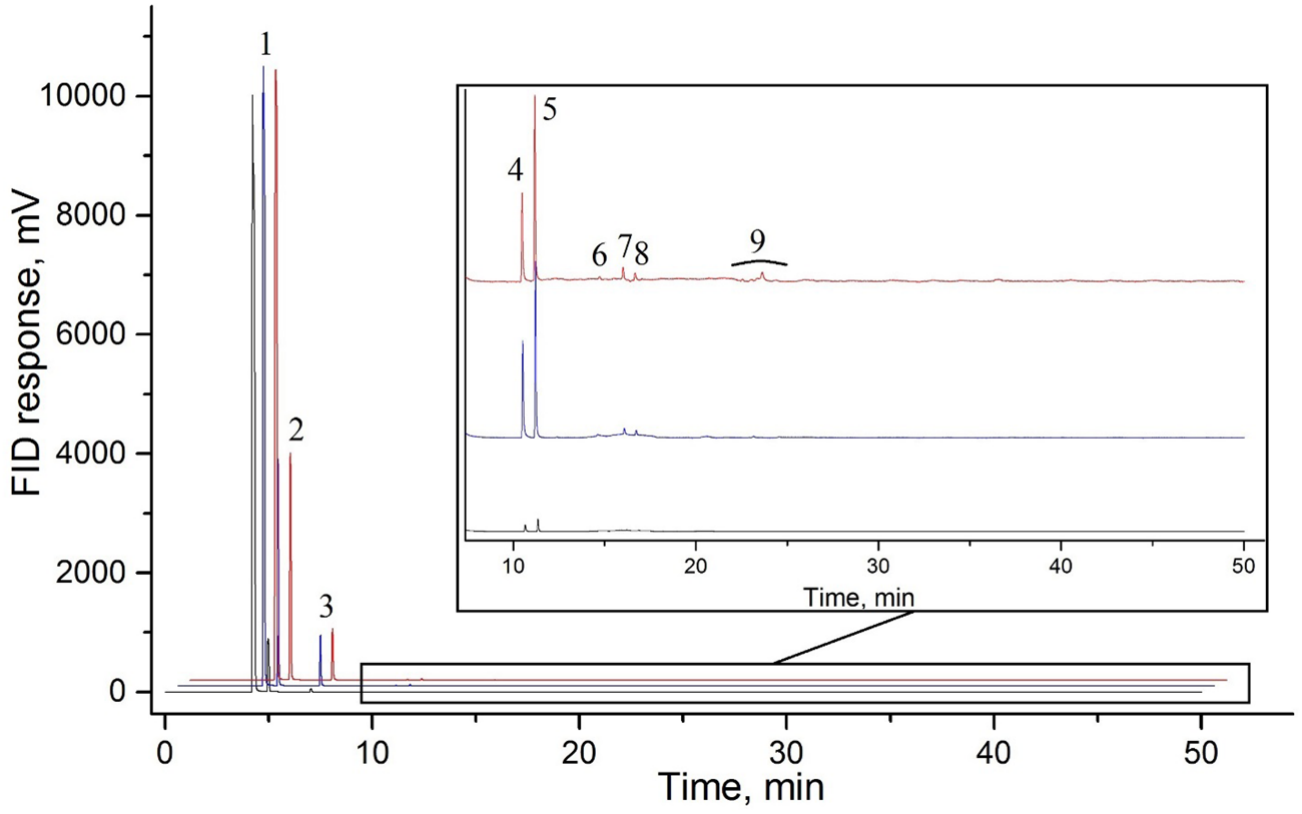
Chromatograms of the hydrocarbon products formed at 1000(±25) K and 2.5(±0.2) GPa during 0.5 hours of heating (black curve), 4 hours of heating (blue curve), 10 hours of heating (red curve). 1 – methane, 2 – ethane, 3 – propane, 4 – i-butane, 5 – n-butane, 6 – neo-pentane, 7 – i-pentane, 8 – n-pentane, 9 – hexanes.

Similar to the first series of experiments, the second series of experiments, carried out at 1000(±25) K and 2.5(±0.2) GPa with 0.5 hours, 4 hours and 10 hours of exposure time, demonstrated the formation of only light saturated hydrocarbons (methane, ethane, propane, n-butane and i-butane) with trace amount of pentane and hexane isomers and unsaturated hydrocarbons (See Supplementary Fig. 5b for more details). The gas chromatogram of the hydrocarbon products is presented in Fig. 3. The fraction composition of the gaseous product mixture is shown in Table 2. Carbon dioxide was not detected in the gaseous products mixture (see Supplementary Fig. 6 for more details). D and G bands of graphite were detected in the mixture of the solid products by Raman spectroscopy (Fig. 2, blue curve)^23^.

**Table 2:** Composition of the products mixture after the heating (1000 K, 2.5 GPa) and of natural gas from Vuktinskoe gas field (for comparison).

Component/fraction, %	*Exposure time, hours*			*Vuktinskoe gas field*
	*0.5*	4	10	
*Methane*	93.495	81.908	75.766	73.800
*Ethane*	6.149	14.808	19.455	8.700
*Propane*	0.336	3.070	3.964	3.900
*i-Butane*	0.008	0.075	0.280	1.800
*n-Butane*	0.012	0.113	0.187	
*C*_5_ *fraction*	—	0.016	0.193	6.400
*C*_6_ *fraction*	—	0.011	0.154	—
*C*_7_ *fraction*	—	—	—	—
*Benzene*	—	—	—	—

## Discussion

The results of the current research at 1000 K are in significant agreement with the results of previous investigations^16,17^. All hydrocarbons produced from methane in DAC by Kolesnikov, *et al*.^17^ were detected in our product mixture synthesized at 1000(±25) K and 2.5(±0.2) GPa: ethane, propane, n-butane and graphite. Iso-butane may also be present in the products mixture in the DAC experiments; however, its detection may be difficult due to the similar Raman signals for propane, butane and i-butane^24,25^. The trace amount of pentane and hexane isomers in our products mixture was detected by virtue of the large volume of the sample and the high sensitivity of the gas chromatography equipment.

The results of the current research support the hypothesis about the “methane path” mechanism of hydrocarbons synthesis from inorganic donors of carbon and hydrogen at extreme thermobaric conditions through the stage of methane formation^11,26^. The abiogenic synthesis of hydrocarbons was carried out in the large high-pressure unit “KONAK” with analysis by gas chromatography. Methane and heavier hydrocarbons were formed from CaCO_3_ and H_2_O in the presence of iron compounds at a wide range of thermobaric conditions (up to 11 GPa and 1800 K). The composition of normal and iso-alkanes up to C_6_H_14_, detected in the product mixture by gas chromatography combined with mass spectrometry, is similar to the hydrocarbon systems, produced from methane in our experiments.

A significant increase in the duration of the heating in our experiments compared to the 10 s exposure of the previous experiments^17,27^ did not drastically change the composition of the reaction products produced at similar pressure and temperature. However, the further increasing in exposure time leaded to the growth of heavy hydrocarbons (pentane and hexane isomers) in the product mixture (Fig. 1). The relative amount of ethane, propane, and butanes was kept almost constant in the series of experiments at 1000 K and 2.5 GPa with 4 hours and 10 hours of exposure time, while the amount of pentane and hexane isomers slightly grew. It contradicts the hypothesis that chemical equilibrium is reached very rapidly, however, the formation of heavier hydrocarbons from methane occurs instantaneously^27^.

**Figure 4:**
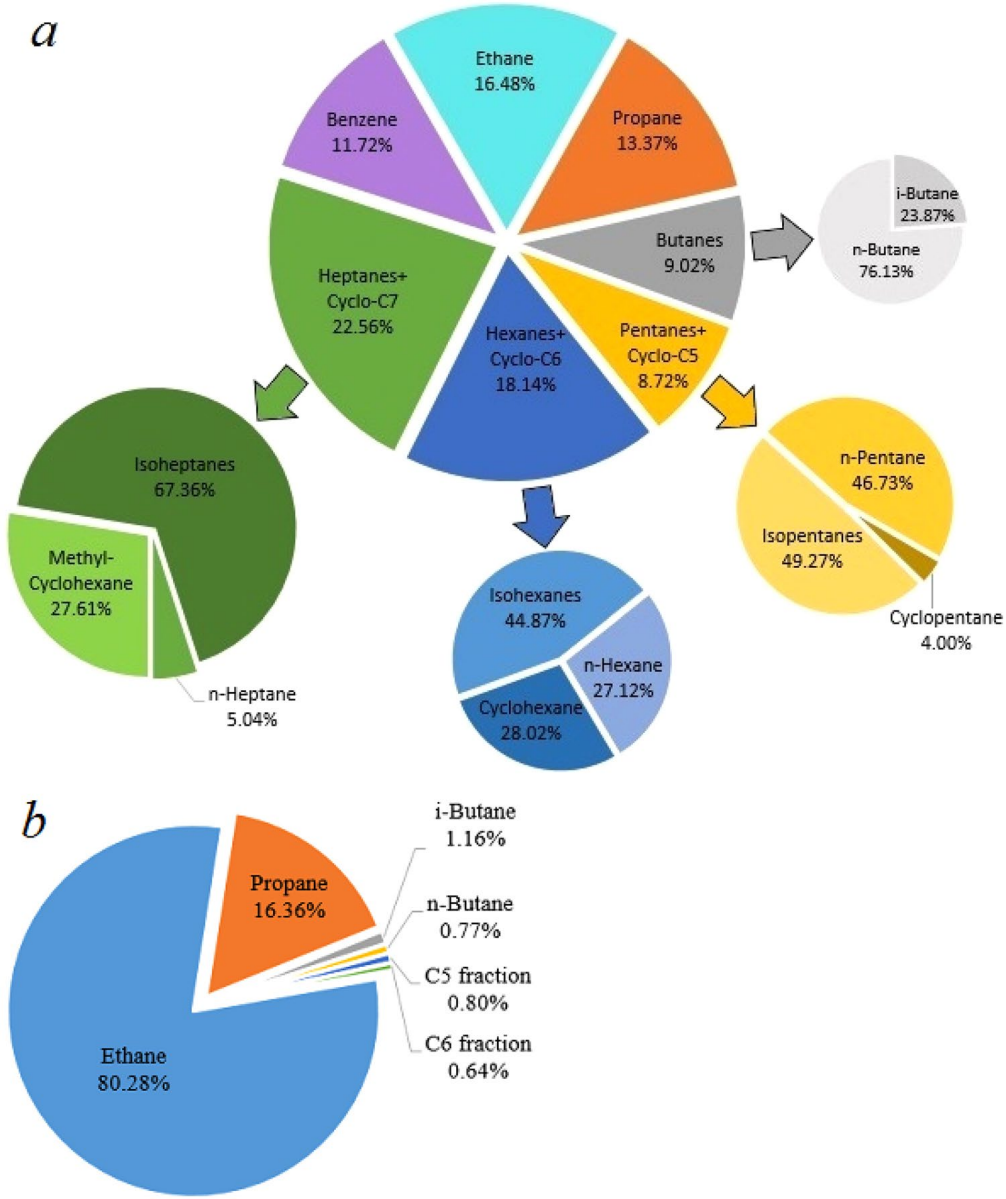
Composition of the heating product mixture (methane excluded): (**a**) formed at 850(±25) K and 2.5(±0.2) GPa during 10 hours, (**b**) formed at 1000(±25) K and 2.5(±0.2) GPa during 10 hours.

The total amount of ethane, propane and butanes is more than 25% volume in the gaseous products synthesized at 1000(±25) K and 2.5(±0.2) GPa, thus making the composition of the “equilibrium” hydrocarbon system similar to “wet” natural gas (Table 2, Fig. 4b).

At a lower temperature (850(±25) K), a complex hydrocarbon mixture (up to seven carbon atoms in composition) was produced from methane. Similar to the series of experiments at 1000(±25) K and 2.5(±0.2) GPa, methane predominated in the product mixture. In addition to the normal alkanes, new classes of hydrocarbons were formed from methane: iso-alkanes, naphthenes and aromatics. All the isomers of alkanes from butane to heptane were detected by gas chromatography.

**Figure 5:**
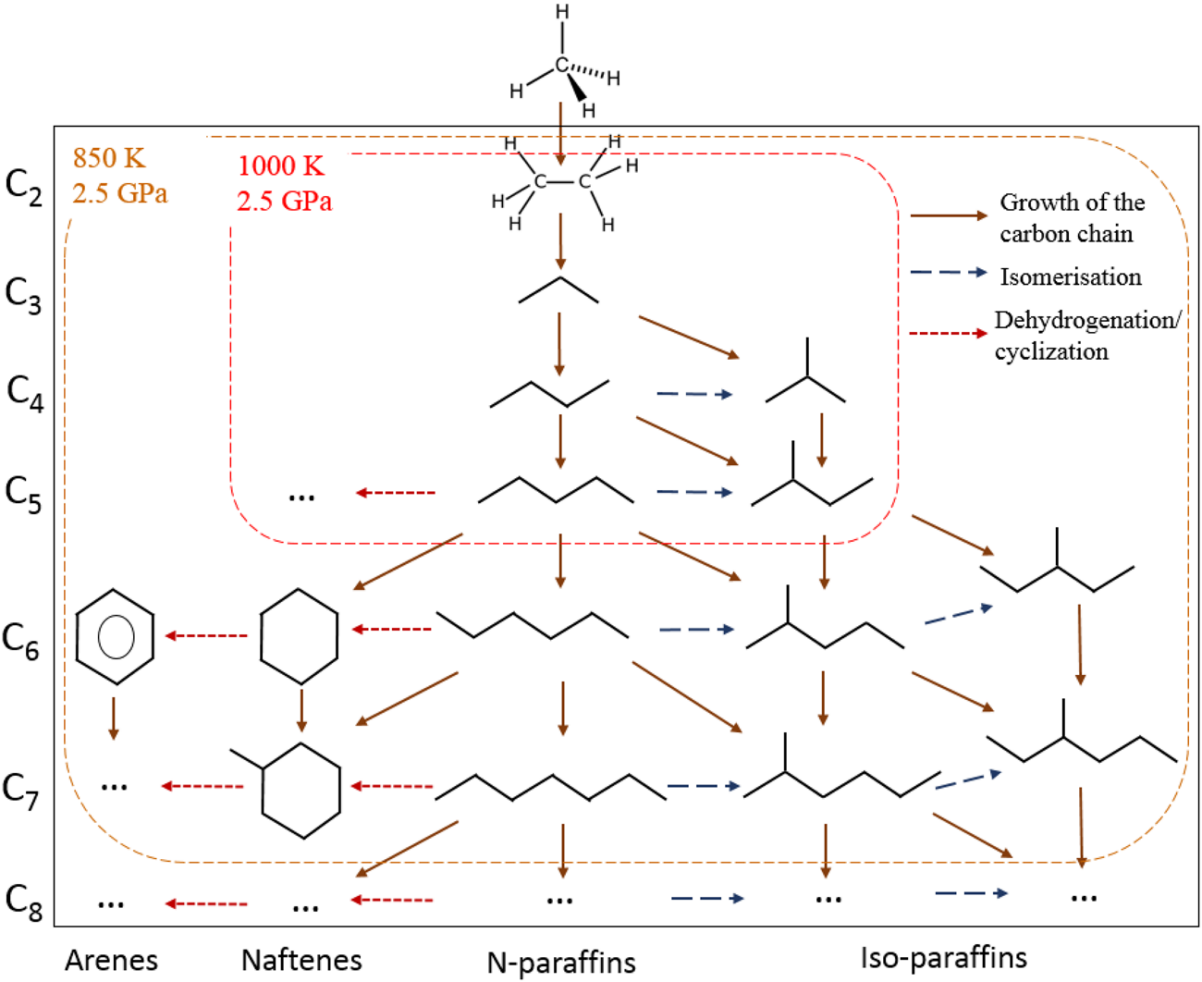
The scheme of methane transformations pathways. Solid brown arrow – reactions with the growth of the carbon-carbon chain, blue dashed line – isomerization of the synthesized hydrocarbon, red dashed line – dehydrogenation with the formation of the cycle chain or aromatic chain.

Figure 4 shows the composition of the gaseous products (methane is excluded) generated from methane after 10 hours of heating at 850(±25) K and 2.5(±0.2) GPa and at 1000(±25) K and 2.5(±0.2) GPa. The product mixture consists of light components of petroleum (Fig. 4a). The scheme of possible pathways of heavier hydrocarbons formation is presented in Fig. 5. The synthesis of heavier hydrocarbons is carried out via the radical mechanism^28^ focused mostly on the growth of the carbon-carbon bonds, isomerization and cyclization. Unsaturated hydrocarbons, which were also detected by Raman spectroscopy in the DAC experiments at similar thermobaric conditions^29^, may be the intermediate components due to their trace amount in the product mixture. One of the possible explanation is the deficiency of hydrogen in the reaction system that may lead to the formation of unsaturated hydrocarbons. In the complex hydrocarbon mixture produced from methane at 850(±25) K and 2.5(±0.2) GPa (Table 1), n-alkanes predominate for butane and pentane fractions in the experiments with time exposure of 0.5 and 2 hours. However, iso-alkanes prevailed in the experiments with more extensive heating (4 and 10 hours) due to the intensification of isomerization reactions^28^. Higher thermal stability of iso-structure can be explained by the more energetically stable and three-dimensionally substantial branched structure of large hydrocarbon molecules. The same situation takes place in the product mixtures produced from methane at 1000(±25) K and 2.5(±0.2) GPa: the relative amount of i-butane increases in the system after 10 hours heating.

Our experiments describe the possible chemical transformations of methane in the C-O-H fluid at thermobaric conditions corresponding to the upper depth border of the abiogenic hydrocarbons formation zone of 70–80 km^20,30^. Methane, generated from the inorganic compounds in this mantle area or transported to this zone from the deeper level of the asthenosphere by the deep fluid^31^ can be transformed into heavier hydrocarbons. The complex hydrocarbon mixtures, generated in the upper mantle from methane, can migrate to the Earth’s crust through deep faults^31^ or in subduction zones along the weakened surface of the slab^32^ and contribute to petroleum deposits.

Our results indicate that at 2.5 GPa the temperature limit for heavier hydrocarbons C_6+_ is somewhere between 850 K and 1000 K. We cannot suggest what are the depth limits of the thermobaric stability zone for complex hydrocarbons mixtures, however, we suppose that at higher pressure the temperature limit for heavier hydrocarbons C_6+_ may be higher. As a result, it is expected that the existence of complex hydrocarbon mixtures is not limited by the depth of 70–80 km, but it is governed by the still unknown pressure-temperature correlation in the mantle.

It was strongly considered that methane was the predominant hydrocarbon component in the mantle fluids, and because of this hypothesis only methane^33,34^ and sometimes methane with ethane^27^) were taken into consideration in the C-O-H the mantle fluid modelling. However, our experiments suggest that a significant part of methane could be transformed into heavier hydrocarbons at the thermobaric conditions of the upper mantle (Tables 1, 2). Therefore, at least in the mantle zones with thermobaric conditions, compatible to ones, modelled in our experiments, it is expected that complex hydrocarbon mixtures may exist and, therefore, should be included in the C-O-H fluid modelling. The possible existence of heavy hydrocarbons in the mantle is supported by the literature data about the hydrocarbon inclusions in the mantle derived xenoliths. The deep mantle xenoliths, observed in various alkaline basic and ultrabasic igneous rocks, are one of the most important sources of information about the nature of the upper mantle. Matson, *et al*.^35^ studied inclusions in amphiboles from the mantle xenoliths selected in Vulcan’s Throne (United States). These amphiboles contain CH_4_, C_2_H_4_, C_3_H_8_, and the heavier hydrocarbons. Methane concentrations vary from 200 to 500 g/t. According to experiments, amphibole-bearing xenoliths crystallize at the depth of 65 km.

## Conclusion

The experimental results obtained suggest that at favorable temperature (1000(±25) K), the components of natural gas (ethane, propane, n-butane and isobutane) can be generated in the C-O-H fluid from methane at the abovementioned depth. In the colder zones of the upper mantle (850(±25) K), a petroleum-like system may be formed. Four major classes of hydrocarbons, which are the basic representatives of natural petroleum (normal alkanes, branched alkanes, naphthenes and aromatic hydrocarbons), may be produced from methane at the mantle moderate thermobaric conditions. The increasing of exposure time during the experiment leads to growth of the amount of heavier hydrocarbons in the product mixture, formed from methane. This fact demonstrates the thermal stability of heavy hydrocarbons at thermobaric conditions, corresponding to the upper mantle.

Due to the novel technique based on the Toroid LRV unit equipped with the gas chromatograph, the methane transformation products were measured quantitatively and qualitatively. The obtained results broaden the existing knowledge about the methane pathway of hydrocarbons formation from inorganic materials^22^ and provide additional information about the possible mechanism of hydrocarbons synthesis from methane at extreme thermobaric conditions. It was shown that at high pressure and temperature, hydrocarbons with the branched structure predominated in the C_5_-, C_6_-, and C_7_-fractions of the reaction products. Future research will be focused on the investigation of this “equilibrium” kinetics and the possible catalytic influence of the mantle components on the hydrocarbon transformation pathways.

## Methods

### High pressure-high temperature Large Reactive Volume (LRV) device “URS-2”

**Figure 6:**
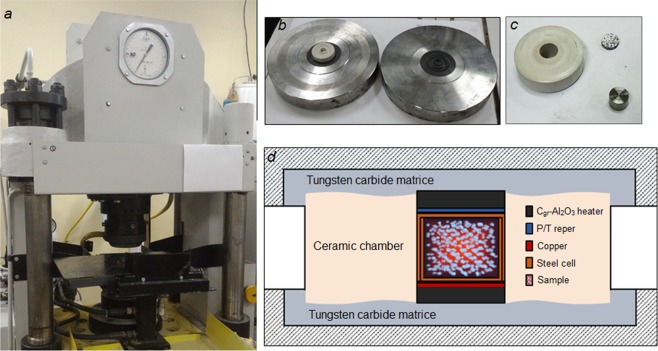
(**a**) “Toroid” Large Reactive Volume (LRV) Device, (**b**) tungsten carbide matrices, (**c**) toroid-shape ceramic container, C_gr_-Al_2_O_3_ heater, steel cylindrical cell, (**d**) the scheme of the sample assembly.

The experiments were carried out in the “toroid-type” large Reactive Volume (LRV) device “URS-2” (designed and manufactured in the Technological Institute of super-hard and novel carbon materials, Troitsk, Russia) (Fig. 6a). The “Toroid” LRV device allows pressures as high as 8 GPa and temperatures as high as 1700 K. The pressure in the unit is caused by the hydraulic system that passes the pressure to the steel cylindrical cell with a diameter of 8 mm and height of 8 mm through a pair of tungsten carbide toroid-shape matrices (Fig. 6b) and the ceramic chamber, serving as the outward pressure medium (Fig. 6c). Heating is performed by passing an alternating electric current through the heaters (made of mixture Al_2_O_3_:C_gr_ as 4:1) placed at the top and bottom parts of the cell (Fig. 6d). Discs made of copper foil were placed between the heater and the cell for additional electrical conductivity. The pressure and temperature in the cell are estimated by the calibration curves, which are preliminarily obtained by taking into account the phase transitions of the reference compounds mounted in the chamber together with the cell during the calibration experiments (Bi, PbTe and PbSe for pressure calibration, Sn, Pb, Ti and Cu for temperature calibration, see^32^ for more details).

The temperature in the cell was increased at a rate of 100 K/min. When the required temperature was reached, it was held during the exposure time (the pressure and temperature inside the sample were controlled automatically by the LRV device managing system).

The “toroid-type” LRV device did not allow direct loading of methane in the cell. Therefore, the loading procedure of methane was replaced by synthesis of methane from aluminum carbide and water directly inside the cell^29^ (see Supplementary Note 1 for more details).

### Sample analysis

When the cell was quenched down to the ambient temperature, the pressure was reduced. The pressure-sealed steel cell was recovered from the misshaped ceramic chamber and mounted in the hermetically sealed gas-extracting camera connected to the gas chromatograph “Chromatech Crystal 5000” (Gubkin Russian state university of oil and gas, Moscow) with an He-carrier and capillary column GS-GasPro (60 m length, 0.32 mm diameter with adsorbed silica gel). The gas chromatograph was equipped with two Flame Ionization Detectors (FID), that allowed examining mixtures of hydrocarbons and inorganic gases. The molar percentage composition of the products mixture components was estimated by an area under the corresponding chromatograph peaks due to an equal response of FID to all components eluted. Analysis of solid reaction products was carried out by Raman spectroscopy (He-Ne laser wavelength 632.8 nm, power 2 mW) using a LabRam spectrometer (2 cm^−1^ spectral resolution).

## Supplementary information

Supplementary Information.

## Data Availability

All data needed to evaluate the conclusions are presented in the paper.
