# Supplementary material for: Formation of complex hydrocarbon systems from methane at the upper mantle thermobaric conditions
Source: Sci Rep. 2020 Mar 12;10:4559. doi: 10.1038/s41598-020-61644-5 (PMC7067895; doi:10.1038/s41598-020-61644-5)
Supplement: Supplementary file 1 — Supplementary Information. [file 41598_2020_61644_MOESM1_ESM.pdf]

Supplementary material for

Formation of complex hydrocarbon systems from methane at the upper mantle thermobaric conditions

Aleksandr Serovaiskii[1*], Vladimir Kutcherov[1]

[1]Gubkin Russian State University of Oil and Gas (National Research University),

Department of Physics, Leninsky avenue 65/1, Moscow, 119991, Russia

*alexandrserov@gmail.com +79096394699

Supplementary Note 1

Synthesis of methane inside the experimental cell.

The powdered aluminum carbide Al_4C_3 (>99%, Sigma Aldrich) was mixed with distilled water at a 1:13 molar ratio (water in excess amount for the complete transformation of the carbide). The Al_4C_3-H_2O mixture was loaded in the cell. The cell with the sample was mounted in the ceramic toroid-shape chamber with the heaters. The assembly was placed in the LRV device between two tungsten carbide matrices. The synthesis of methane (reaction (1)) from the aluminum carbide-water mixture (water in excess amount) was carried out at 473(±25) K and 2(±0.2) GPa.

$$Al_4C_3 + 6H_2O \rightarrow 3CH_4 + 2Al_2O_3 \quad (1)$$

The synthesis was conducted during a 30-minute period; then, the heating was stopped, and the sample was quenched down to ambient temperature and decompressed. The chromatogram of the gaseous products is presented in Supplementary Fig. 1. The synthesized gas mixture consisted of 99.26% methane with the impurities of ethane (0.11%), propane (0.35%), n-butane (0.05%), i-butane (0.16%) and trace amount of light unsaturated hydrocarbons (ethylene, propylene) and pentane isomers. Raman spectra of the solid part of the reaction products demonstrated the absence of Al_4C_3 in the mixture and the formation of Al_2O_3 (Supplementary Fig. 2, black curve), confirming the complete conversion of aluminum carbide.

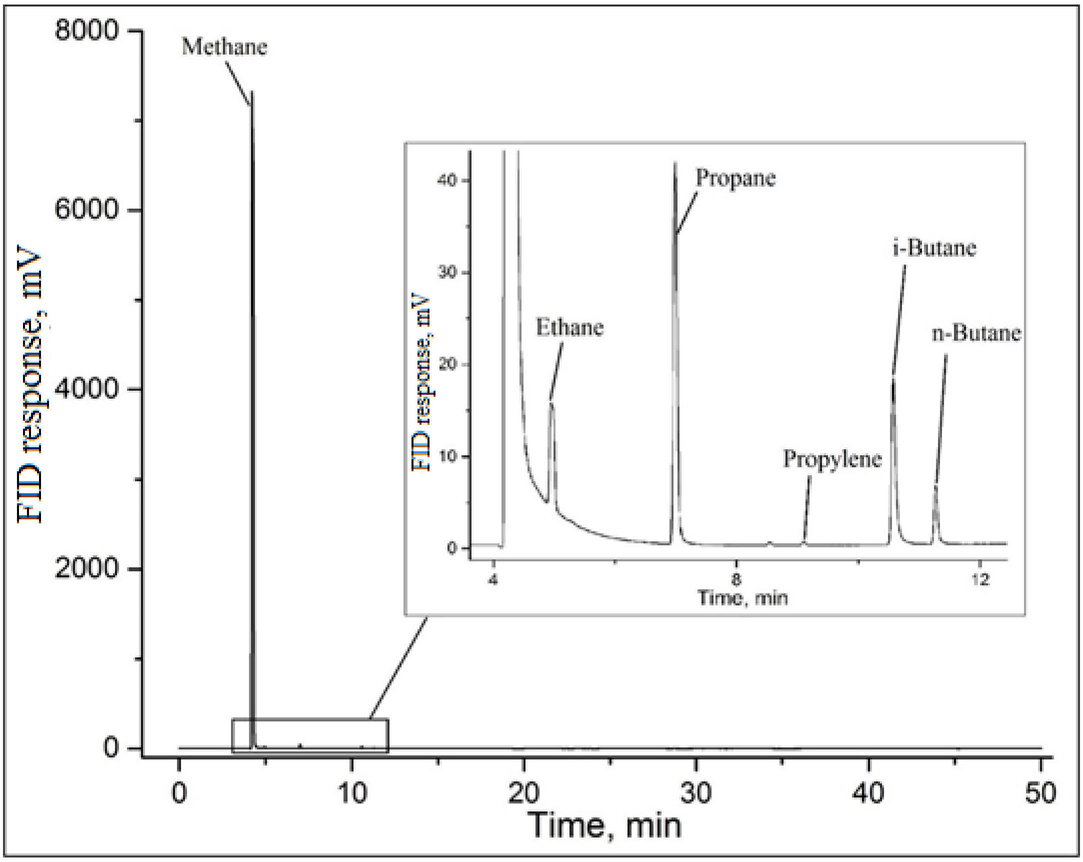

Supplementary Fig. 1. Chromatogram of the synthesized methane with impurities, formed at 473(±25) K and 2(±0.2) GPa during 30 minutes of heating.

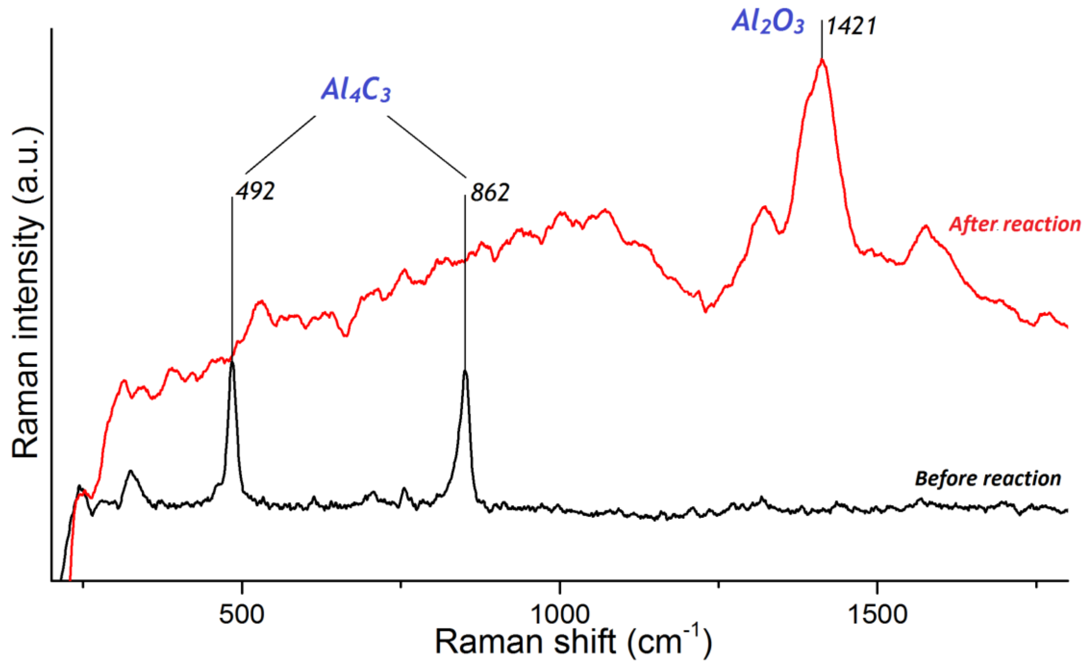

Supplementary Fig. 2. Raman spectra of the sample before the reaction at ambient conditions (black curve) and the solid products, formed at 473(±25) K and 2(±0.2) GPa, at ambient conditions (red curve).

Therefore, all series of experiments in our investigation started with the step of the methane synthesis from the mixture of aluminum carbide and water inside of the experimental cell at 473(±25) K and 2(±0.2) GPa during a 30-minute period with the subsequent increase in pressure and temperature without recovering the cell from LRV.

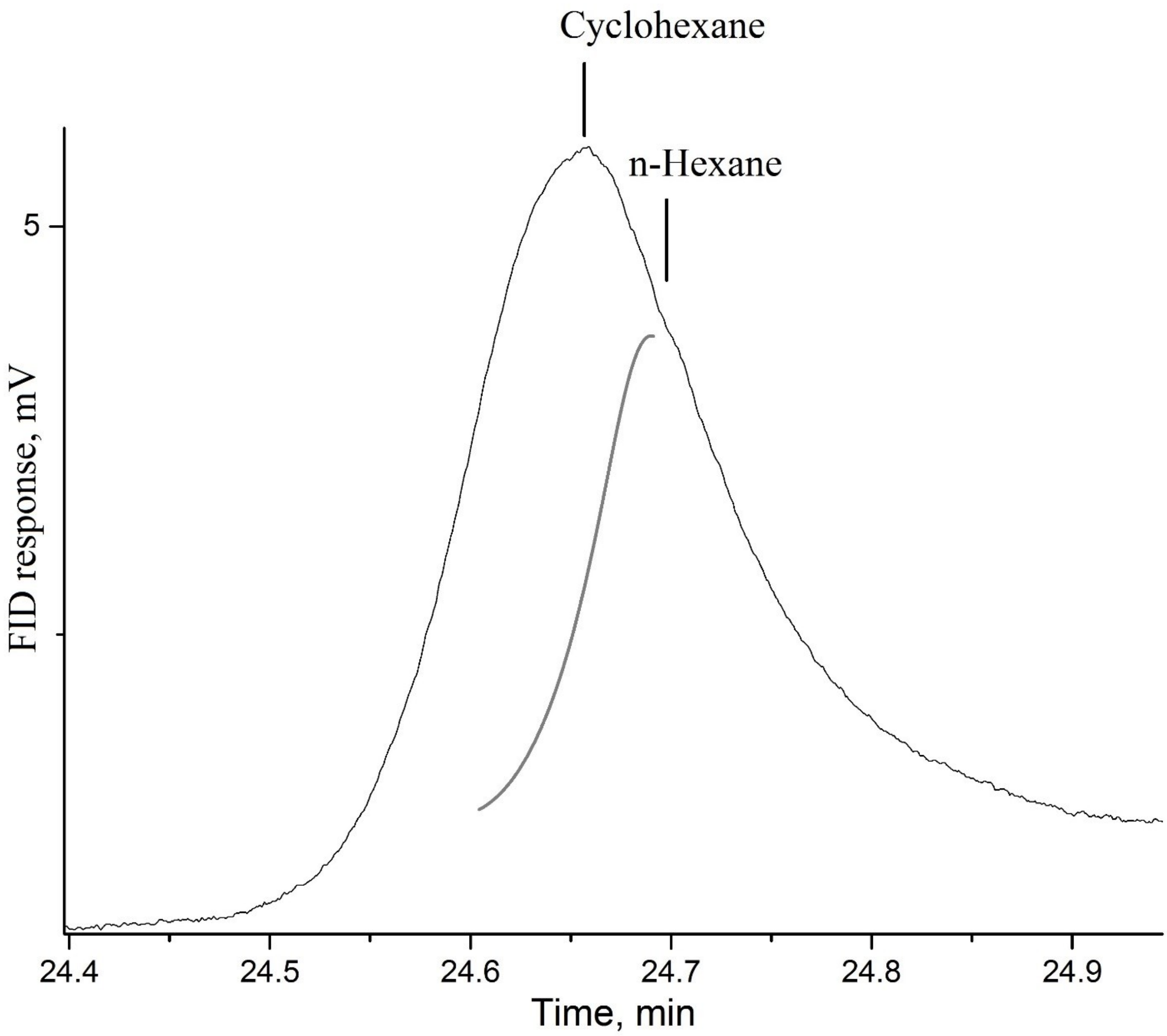

Supplementary Fig. 3. Peaks of cyclohexane and n-hexane at the chromatogram of the hydrocarbon products, formed at 850(±25) K and 2.5(±0.2) GPa during heating for 4 hours.

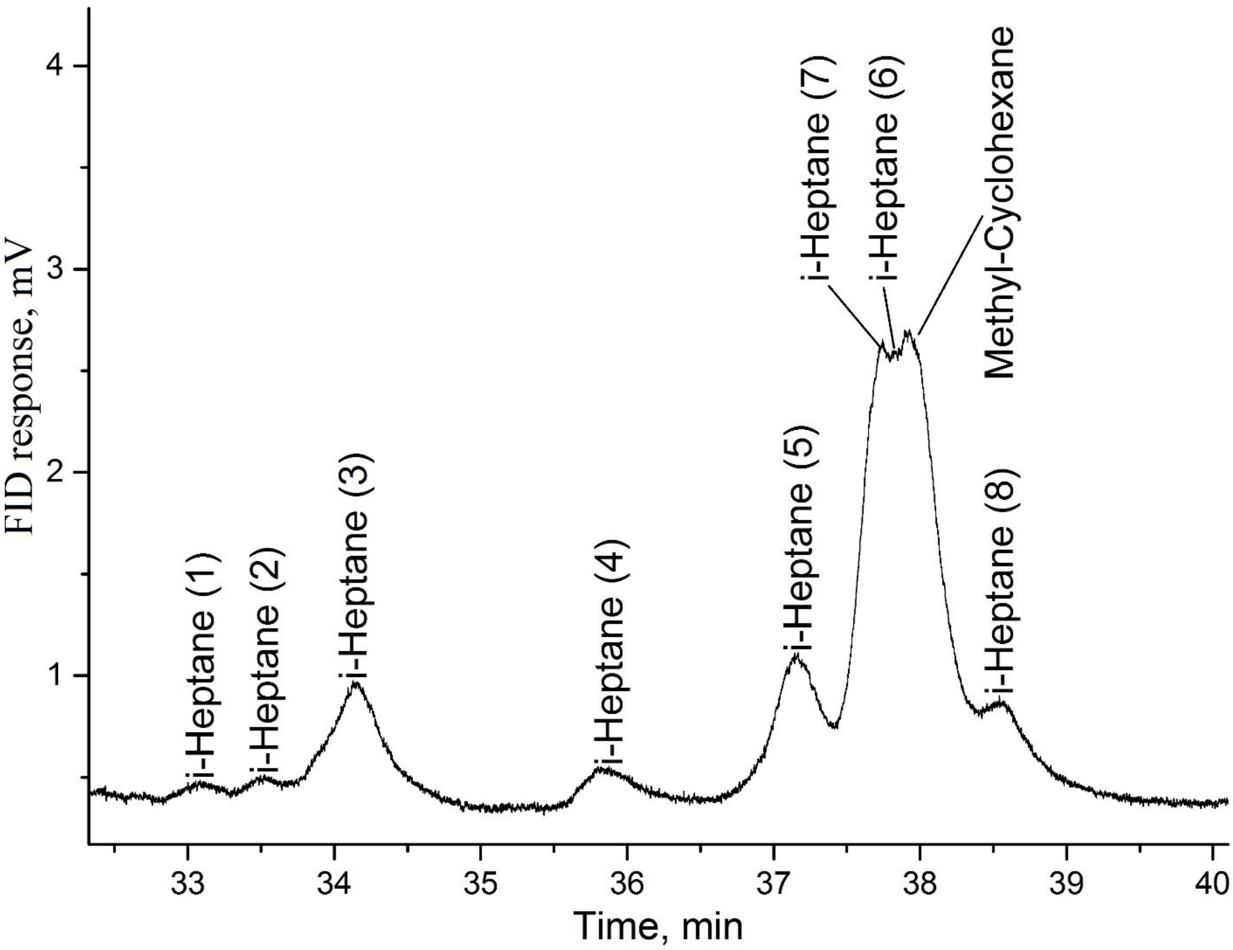

Supplementary Fig. 4. Peaks of methyl-cyclohexane and isoheptanes at the chromatogram of the hydrocarbon products, formed at 850(±25) K and 2.5(±0.2) GPa during heating for 4 hours.

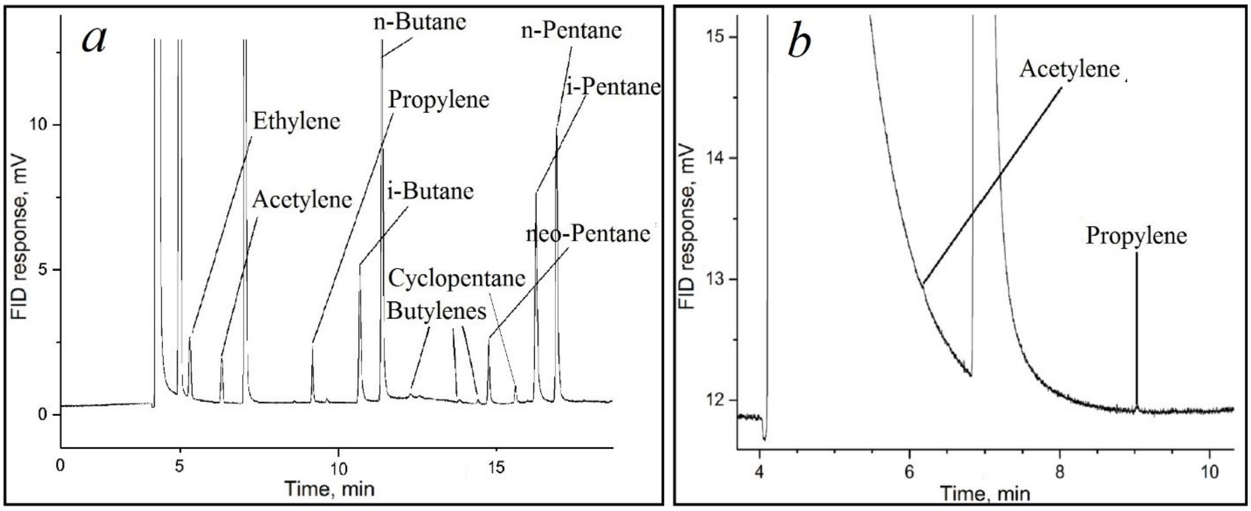

Supplementary Fig. 5. Peaks of light unsaturated hydrocarbons: a – chromatogram of the hydrocarbon products, formed at 850(±25) K and 2.5(±0.2) GPa during heating for 4 hours; b – chromatogram of the hydrocarbon products, formed at 1000(±25) K and 2.5(±0.2) GPa during heating for 4 hours.

CO_2 detection in gaseous products

Carbon dioxide content in the product mixture was estimated relatively to the CO_2 content in air. The calibration chromatogram was made before each measurement. The data obtained from the calibration measurement was subtracted from the products chromatogram (Supplementary Figure 3).

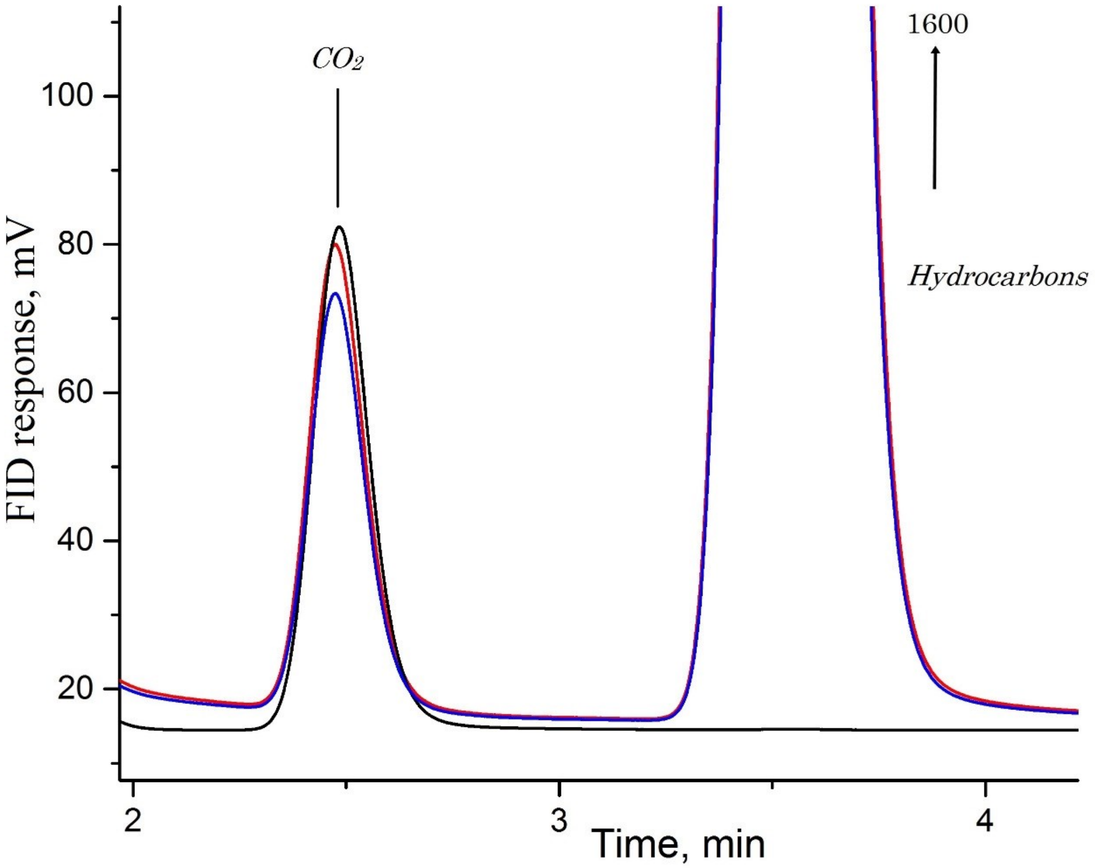

Supplementary Fig. 6. Chromatogram of the gaseous products of methane transformation: black curve – calibration curve of the air in the gas-extracting camera, red curve – after heating at 850(±25) K and 2.5(±0.2) GPa, and the solid products, blue curve – after heating at 1000(±25) K and 2.5(±0.2) GPa.
